# Assessment of the validity and reliability of the 32-item Motor Function Measure in individuals with Type 2 or non-ambulant Type 3 spinal muscular atrophy

**DOI:** 10.1371/journal.pone.0238786

**Published:** 2020-09-18

**Authors:** Dylan Trundell, Stephanie Le Scouiller, Laure Le Goff, Ksenija Gorni, Carole Vuillerot

**Affiliations:** 1 Roche Products Limited, Welwyn, United Kingdom; 2 Department of Pediatric Physical Medicine and Rehabilitation, Hôpital Mère Enfant, CHU-Lyon, Lyon University, Lyon, France; 3 PDMA Neuroscience and Rare Disease, F. Hoffmann-La Roche Ltd, Basel, Switzerland; IRCCS E. Medea, ITALY

## Abstract

The 32-item Motor Function Measure (MFM32) is an assessment of motor function, and its measurement properties were established in a broad neuromuscular disease population. This study sought to investigate the reliability, validity, and ability to detect change of MFM32 in individuals with Type 2 and non-ambulant Type 3 spinal muscular atrophy (SMA). Data were used from the Phase 2 study assessing the efficacy and safety of olesoxime. A total of 110 individuals with Type 2 or 3 SMA were included in the analyses. Test-retest reliability (intraclass-correlation coefficient in global impression-defined stable individuals), internal consistency (Cronbach’s alpha), convergent validity (Spearman rank order correlations with other measures), known-groups validity (analysis of covariance comparing Hammersmith Functional Motor Scale -defined groups), and ability to detect change (analysis of covariance comparing global impression-defined groups) were calculated. Strong evidence of test-retest reliability (intraclass-correlation coefficient = 0.93–0.95), internal consistency (Cronbach’s alpha = 0.89), convergent validity (Hammersmith Functional Motor Scale: rho = 0.87; forced vital capacity: rho = 0.61), known-groups validity (all *p*<0.0001), and ability to detect change (all *p*<0.001) were demonstrated. These results provide evidence of the MFM32’s measurement properties, supporting its use in longitudinal research in individuals with Type 2 and non-ambulant Type 3 SMA.

## Introduction

Spinal muscular atrophy (SMA) is a rare and severe progressive neuromuscular disease that causes muscle atrophy and disease-related complications which affect the whole body [1, 2]. SMA is caused by reduced levels of the survival of motor neuron (SMN) protein due to deletions and/or mutations of the *SMN1* gene [3]. A second paralogue gene, *SMN2*, produces low levels of functional SMN protein that are not sufficient to fully compensate deficiency due to the lack of an *SMN1* gene [4–6]. SMA manifests in various degrees of severity, all of which exhibit progressive muscle weakness and mobility impairment [7].

The clinical presentation of SMA ranges in disease severity and affects infants through adults. Rather than one single, well-delineated phenotype, SMA represents a spectrum of motor and functional disabilities, characterized by age of onset and highest motor milestone achieved. Patients are typically categorized into four main subtypes [8, 9]. SMA Types 2 and 3, the focus of this study, reflect a continuum of motor function impairment from individuals with poor fine motor function to individuals who are still ambulant, including overlap between stronger Type 2 and non-ambulant Type 3 individuals. Type 2 SMA usually manifests between 6 and 18 months of age with patients unable to walk without support [8, 10, 11]. Type 3 SMA is less severe, typically manifesting after 18 months of age, with patients achieving the ability to stand and walk without support until the disease progresses [10, 11]. The level of gross motor function development achieved varies across Type 2 and 3 SMA individuals, and is followed by progression, removing and limiting previously acquired function.

Valid and reliable measures of SMA-related outcomes allow for a better understanding of the natural history of all SMA types and of subgroups of disease trajectory [12–14]. Additionally, assessments which are sensitive to change and measure symptoms and outcomes that are meaningful to individuals with SMA are essential for assessing therapeutic strategies to treat SMA. Due to a heterogenous patient population, in terms of age and clinical phenotype, measures of treatment outcomes in SMA must be able to detect changes across the disease spectrum for which they are intended to be used [15].

Among them, the 32-item Motor Function Measure (MFM32) is an assessment of motor function for use in individuals with neuromuscular disease. Reliability and validity of the MFM32 have been demonstrated in a group of individuals with neuromuscular disorders [16]. The MFM32 has been assessed in an SMA population, with evidence of convergent validity (strongest associations with measures of strength and upper limb function) and known-groups validity (discriminating between sitters and non-sitters, and between ambulant and non-ambulant individuals) demonstrated [17]. Additionally, preliminary evidence of responsiveness was demonstrated in a small sample of SMA Type 2 (n = 12) and Type 3 (n = 19) individuals [18]. Furthermore, the MFM32 has been shown to be targeted to assessing individuals across the non-ambulant spectrum [19]. Although fit to the Rasch model was suboptimal in that study, this is due to the multidimensionality of the MFM32. This was highlighted by a more recent analysis in patients with neuromuscular disorders, which demonstrated good fit to a three-dimension confirmatory factor analysis model [20].

Despite these studies, there remains a gap in understanding the measurement properties of the MFM32 in an SMA population (e.g., reliability and ability to detect change). This is particularly important in the context of ongoing clinical research. For example, MFM32 score is the primary efficacy outcome assessment in SUNFISH (NCT02908685), a placebo-controlled, randomized study assessing the efficacy and safety of risdiplam in individuals with Type 2 and non-ambulant Type 3 SMA. Understanding the measurement properties of the MFM32 is critical for interpreting the results of this and future clinical trials that use the assessment scale.

The aim of this study is to assess the measurement properties of the MFM32 in a Type 2 and non-ambulant Type 3 SMA population. A population similar to SUNFISH was studied in the Phase 2, adaptive, parallel-group, double- blind, randomized, placebo-controlled, multicenter, multinational study designed to assess the efficacy and safety of olesoxime over 24 months in 3 to 25-year-old individuals with Type 2 and non-ambulatory Type 3 SMA (NCT01302600). Such datasets provide convenient samples for assessing measurement properties of the scales included. The purpose of this analysis was to retrospectively investigate the validity, reliability, and ability to detect change, of the MFM32 in children, adolescents and young adults with Type 2 and non-ambulant Type 3 SMA, using data from the Phase 2 study.

## Material and methods

### Ethics

For this study, all patients or their parent or guardian provided written informed consent before screening. The study was approved by local institutional review boards and ethics committees including Federal Institute for Drugs and Medical Devices (BfArM; Germany), Rejestracji Produktów Leczniczych Wyrobów Medycznych i Produktów Biobójczych (Poland), Division R&D Federal Agency for Medicines and Health Products (FAMHP; Belgium), Central Committee on Research Involving Human Subjects (CCMO; Netherlands), Medicines and Healthcare Products Regulatory Agency (United Kingdom), The French Health Product Safety Agency (AFSSAPS; France), and the Ethics Committee for Medical Experimentation at the Ospedale Pediatrico Bambino Gesù (Italy). Informed consent was obtained prior to any study-related procedure.

### Analysis population

A total of 165 patients (randomized 2:1 to daily, oral 10 mg/kg olesoxime or placebo) were recruited in Belgium, France, Germany, Italy, the Netherlands, Poland, and the UK. The screening visit included a standard clinical examination, biological tests and SMA status assessments which were part of the routine care. The double-blind treatment period lasted 104 weeks [21]. Key inclusion criteria [21] were weakness and hypotonia consistent with a clinical diagnosis of Types 2 or 3 SMA, genetic confirmation of SMA, an MFM32 relative score (percentage of the maximum sum of both dimensions) ≥15% (D [domain] 1+D2 score), a Hammersmith Functional Motor Scale (HFMS) score at baseline between 3 and 38, age at onset of symptoms ≤3 years of age, and the ability to take the study treatment (tested at screening after informed consent). Key exclusion criteria [21] included diagnosis of a neurodegenerative or neuromuscular disease other than SMA and participation in any other investigational drug or therapy study within the previous 3 months. Further details of the Phase 2 study are reported by Bertini, et al. (2017) [21]. For this analysis, only patients with available MFM32 data (as appropriate for each analysis) were included. As participants less than 6 years old were administered an abbreviated version of the MFM 20 items (the MFM20), the analysis only included individuals aged 6 years and older. This resulted in an analysis sample of 110 patients.

### Outcome assessments

The following outcome assessments were included in the analysis. Further details can be found in Bertini, et al. (2017) [21].

### MFM

The MFM32 was used to assess motor function in patients. The 32 items of the MFM32 are distributed across three domains: D1 measures standing, transfers and ambulation, D2 measures proximal and axial function and D3 measures distal function. A 4-point Likert scale was used to rate the participant’s maximal ability without assistance: 0, does not initiate movement or starting position cannot be maintained; 1, partially completes the exercise; 2, completes the exercise with compensations, slowness or obvious clumsiness; 3, completes the exercise with a standard pattern. Scoring was conducted in line with the MFM 2^nd^ edition user manual (2009). The raw sum score of the 32 items was converted to a 0–100 scale (by dividing by the total possible raw score of 96 and multiplying by 100). The developers created this scoring to aid interpretation of the score as a ‘percentage’ of normal function. The MFM32 was completed at baseline, and at Weeks 26, 52, 78 and 104. Physiotherapists were trained and certified in the administration of the MFM32.

### HFMS

Motor function was also measured using the HFMS scale of 20 items. Each activity (item) was scored on a 3-point scoring system, with a score of 2 for unaided, 1 for assistance and 0 for inability [22]. Total scores, achieved by summing the scores for all the individual items, range from 0 to 40, with lower scores indicating more severe motor impairment (as defined in the user manual). The HFMS was completed at screening, and Weeks 13, 39, 65, and 91. Physiotherapists were trained and certified in the administration of the HFMS.

A major source of variation between the MFM32 and the HFMS is the presence of distal function items in the MFM32, permitting gradation of severity in weak Type 2 patients. Indeed, a floor effect has been noted on the HFMS [23]. Data from screening were used in the analysis.

### Forced Vital Capacity (FVC)

Pulmonary function was assessed by measuring FVC as a percentage of that which was predicted for age and height. To adjust the FVC according to individual height, weight and gender, the FVC results were divided by the theoretical capacity, and calculated as a percentage. Theoretical capacity was calculated using the following approach (age: years; height: meters):

- For females aged <18 years: 1.4507 + (1.48 + 0.0127*Age)*Height [24].
- For males aged <18 years: 1.2782 + (1.3731 + 0.0164*Age)*Height [24].
- For females aged >18 years: 4.43*Height—0.026*Age—4.34 [25].
- For males aged >18 years: 5.76*Height—0.026*Age—4.34 [25].

Where height could not be measured (e.g., with scoliosis or contractures), ulna length was used to calculate a surrogate height measure [26]. FVC was assessed at baseline and Weeks 13, 26, 39, 52, 78, and 104.

Muscle atrophy is known to impact both motor function and respiratory function, making FVC a useful assessment for convergent validity analysis. Data from baseline were used in the analysis.

### Global impression of change (GI-C)

The GI-C scale was used to assess change from baseline in the patient’s global health using a 7-point ordinal scale: 1-very much improved; 2-Much improved; 3-Minimally improved; 4-No change; 5-Minimally worse; 6-Much worse; 7-Very much worse [27]. A version was completed by the clinician (Clinician GI-C [CGI-C]) and another version by either the patient or a parent (Patient/Parent GI-C [PGI-C]) at each post-baseline visit.

### Analyses

The analyses to assess the measurement properties of the MFM32 were conducted in patients ≥6 years old who were administered the MFM32. Data were pooled across treatment arms for all analyses. Some analyses used a subset of patients, as defined by the analysis (e.g., test-retest reliability) or by the availability of data (e.g., patients without follow-up data were not included in ability to detect change analyses). Item level missing data for study endpoints were handled according to the respective manuals (for example, missing item scores were set to 0 [unable to perform the task] prior to the calculation of the total score. SAS v9.2 was used for all analyses. For analyses that require a statistical comparison of groups (e.g., known-groups validity), the threshold for statistical significance was *p*<0.05. No adjustments for multiplicity were made. For analyses identifying relationships between variables (e.g., internal consistency), suggested values of acceptability were used to aid interpretation. Tests for normality were conducted (e.g., Shapriro–Wilk and Kolmogonov–Smirnov). All results were non-significant supporting the use of the parametric tests described below.

### Sociodemographic descriptive statistics

Sociodemographic data were collected in order to characterize the patient sample. Baseline descriptive statistics (frequency and percentage for categorical variables; mean, standard deviation [SD], minimum and maximum for continuous variables) were calculated for gender, age (at informed consent), country, SMA type, and MFM32 score at baseline.

### Reliability

#### Test-retest reliability

Test-retest reliability assesses the stability of a score in patients who are not expected to have experienced a change in the construct of interest. Whilst test-retest reliability is often assessed over short durations, the approach does not give confidence in the reliability of a measure that is used over long-duration studies. Test-retest reliability of the MFM32 total score was assessed by calculating the intraclass correlation coefficient-model 2,1 (ICC2,1): a two-way random effects analysis of variance (patient by visit), in a subset of individuals classified as stable. Stable was defined using two different anchors (with separate analyses conducted for each group): 1) patients classified as “no change” on the CGI-C at Week 26; and 2) patients classified as “no change” on the PGI-C at Week 26. This methodology is commonly used, and is recommended by COnsensus‐based Standards for the selection of health Measurement Instruments (COSMIN) initiative [28, 29]. ICCs≥0.7 were considered acceptable [30].

#### Internal consistency reliability

Internal consistency reliability assesses the homogeneity of items belonging to the same scale or domain [31]. Internal consistency of the MFM32 was assessed by calculating Cronbach’s alpha at baseline. Values ≥0.7 were considered acceptable [30, 31].

### Validity

#### Convergent validity

Convergent validity is an assessment of the relationship between the target measure (in this case the MFM32) and related measures. Stronger correlations should be demonstrated with more related constructs. Convergent validity of MFM32 total score was assessed via Spearman rank order correlations with HFMS score (HFMS at screening, MFM32 at baseline) and with FVC (at baseline). To aid interpretation, the following thresholds were used [32, 33]: <0.2: Weak; ≥0.2 to <0.4: Modest; ≥0.4 to <0.6: Moderate; ≥0.6 to 0.8: Strong; ≥0.8: Very strong. Conceptually, the HFMS measures the most related construct to the MFM32 (i.e., both the measures assess motor function ability), and thus the strongest correlation was expected with this measure. Respiratory function is known to be impacted in this population, so the relationship was expected to be relatively strong between FVC and the MFM32; however, it was not expected to be as strong as the correlation between the MFM32 and the HFMS.

#### Known-groups validity

The validity of a measure can be assessed by its ability to discriminate between two or more groups that are known to be different. For example, a measure of motor function should produce statistically significantly different scores for patients that are known to have greater function than those who are known to have lower levels of function. The level of function can be defined by a related measure. In this study, groups were defined by:

- HFMS score at screening: ≥ median vs < median
- ability to stand: able to stand vs unable to stand
- ability to sit with legs straight and back unsupported: able to sit vs unable to sit.

Ability to stand was assessed by Items 18 and 19 of the HFMS at screening. Unsupported standers, at screening, had an HFMS score of:

- 2 on Q18 (stand with support of one hand), AND
- 1 or 2 on Q19 (unsupported for count of 3, or unsupported for count >3).

Supported standers, at screening, had an HFMS score of:

- 1 (stand with minimal trunk support) or 2 on Q18, AND
- 0 on Q19 (stand <3 or unable).

Non-standers, at screening, had an HFMS score of:

- 0 on Q18 (requires knee/hip support or unable), AND
- 0 on Q19.

Supported and unsupported standers were grouped together due to small sample sizes.

Ability to sit was assessed by Item 2 of the HFMS at screening.

- Able to sit without support is defined by HFMS item 2 score = 2.
- Unable to sit without support is defined by HFMS Item 2 score <2.

Known-groups validity was assessed by comparing mean MFM32 total scores for each group via analysis of covariance (ANCOVA; controlling for age and gender) at baseline. A significant difference (*p*<0.05) between the groups provides evidence of known-groups validity.

### Ability to detect change

Ability to detect change can be assessed by evaluating differences in the target outcome assessment’s change in scores between groups that are known to have had a different longitudinal outcome. It is an evaluation of the measure’s ability to detect differences, not an evaluation of the measure’s ability to detect a treatment effect (the cause of the groups’ difference is not what is being assessed but rather the responsiveness of the scale). Thus, even natural history datasets with untreated individuals could be used (i.e., the outcome of the Phase 2 study is largely irrelevant for this analysis). Ability of the MFM32 to detect change was evaluated comparing change from baseline to Week 104 scores in ‘responders’ vs ‘non-responders’ via ANCOVA (controlling for baseline MFM32 score, age and gender). Analyses were conducted using two different anchors to define responders and non-responders (separate analyses). For both CGI-C and PGI-C, responders were defined as those scored as no change, minimally improved, much improved or very much improved, and non-responders were those scored as minimally worse, much worse, or very much worse. A significant difference (*p*<0.05) between the groups provides evidence of ability to detect change.

## Results

### Patient demographics

Baseline patient demographics are listed in Table 1. Despite the intended upper age cap in the Phase 2 study of 25 years, one individual aged 27 years was enrolled in the study. As the purpose of our analysis was to assess the measurement properties in children, adolescents and young adults, this individual was included in the analysis, resulting in a mean age of 12 years (6–27 years). More than half of the sample were male (56%), and most had SMA Type 2 (62%). Baseline MFM32 scores ranged from 21.9–69.8, providing a broad range of functional ability.

**Table 1:** Patient demographic characteristics at baseline.

Characteristic	All patients (N = 110)	Type 2 patients (N = 68)	Type 3 patients (N = 42)
Age, years			
Mean (SD)	12.4 (5.2)	11.2 (4.9)	14.4 (5.0)
Min-Max	6–27	6–27	6–25
Gender, n (%)			
Male	62 (56%)	40 (59%)	22 (52%)
Female	48 (44%)	28 (41%)	20 (48%)
SMA Type, n (%)			
2	68 (62%)		
3	42 (38%)		
Country, n (%)			
Belgium	8 (7%)	7 (10%)	1 (2%)
France	18 (16%)	14 (21%)	4 (10%)
Germany	12 (11%)	8 (12%)	4 (10%)
Italy	37 (34%)	24 (35%)	13 (31%)
Netherlands	7 (6%)	7 (10%)	0 (0.00%)
Poland	17 (15%)	2 (3%)	15 (36%)
United Kingdom	11 (10%)	6 (9%)	5 (12%)
MFM32			
Mean (SD)	47.3 (10.8)	43.4 (9.4)	53.7 (9.9)
Min-Max	21.9–69.8	21.9–63.5	32.3–69.8

MFM32, 32-item Motor Function Measure; SD, standard deviation; SMA, spinal muscular atrophy.

Evaluation of the impact of missing MFM32 item scores at baseline indicated that majority of patients (97%) had no missing data. The three remaining patients (3%) missed 1, 9 and 11 items respectively. For the two patients with multiple missing item data, the items missing were the more challenging tasks including ambulation assessments. Imputing a score of 0 for these items was consistent with expectations based on other item scores. Similar levels of missing data were seen at Weeks 26 (3%) and 104 (6%). Thus, any impact of missing data and imputation is limited due to the high completion rate.

### Test-retest and internal consistency reliability

All reliability analyses achieved the acceptable threshold of >0.7 for the respective tests. Specifically, an ICC of 0.93 was found in patients classified as “no change” on CGI-C (N = 92), and an ICC of 0.95 in patients classified as “no change” on PGI-C (N = 74), and Cronbach’s α = 0.89.

### Convergent validity

As expected, the correlation between the MFM32 and the HFMS (Spearman’s ρ = 0.87, *p*<0.0001, N = 110) was greater than that between the MFM32 and the FVC (Spearman’s ρ = 0.61, *p*<0.0001, N = 104).

### Known-groups validity

Table 2 reports the results of the known-groups validity analyses. Least square (LS) means followed expected patterns (i.e., larger MFM32 total scores for less severe patients) for HFMS score at screening (≥11 vs <11), ability to stand, and ability to sit (LS mean difference = 17.4, 16.5 and 11.9, respectively). All analyses demonstrated significant differences (*p*<0.0001) between groups.

**Table 2:** Known-groups validity: ANCOVA comparing groups’ MFM32 scores at baseline.

**HFMS**	**F (DF)**	**N**	**MFM32 LS Mean**	**MFM32 LS Mean Difference**
<median	61.7* (3, 106)	54	38.6	17.4*
≥median		56	56.0	
**Standing status**	**F (DF)**	**N**	**MFM32 LS Mean**	**MFM32 LS Mean Difference**
Cannot stand	15.3* (3, 106)	93	44.9	16.5*
Can stand		17	61.4	
**Sitting Status**	**F (DF)**	**N**	**MFM32 LS Mean**	**MFM32 LS Mean Difference**
Sit with support	13.6* (3, 106)	41	39.8	11.9*
Sit without support		69	51.7	

*p<0.0001. DF, degrees of freedom; HFMS, Hammersmith Functional Motor Scale; LS, least squares; MFM32, 32-item Motor Function Measure.

### Ability to detect change

As shown in Table 3, the MFM32 was able to detect a change in global condition as assessed by both clinician-rated and patient-/parent-rated change in global health. Significant differences were found between groups’ LS mean change scores (*p*<0.001), using both anchors, with MFM32 change scores following logical patterns (i.e., worsening in the “minimally worse” or worse groups, and small improvements in the “no change” or better groups). Of note, similar LS mean values were similar for both CGI-C- and PGI-C-defined groups, with a decline of approximately 5 points in the worsening groups, and an improvement just below 1 point in the stable/improving groups.

**Table 3:** Ability to detect change: ANCOVA comparing groups’ MFM change from baseline to Week 104 scores.

**CGI-C**	**F (DF)**	**N**	**MFM32 LS Mean Change**	**MFM32 LS Mean Change Difference**
Minimally worse or worse	4.3* (4, 93)	13	-5.3	6.1**
No change or better		85	0.8	
**PGI-C**	**F (DF)**	**N**	**MFM32 LS Mean Change**	**MFM32 LS Mean Change Difference**
Minimally worse or worse	4.7* (4, 93)	14	-5.4	6.3**
No change or better		84	0.9	

*p<0.01

**p<0.001.

CGI-C, Clinician Global Impression of Change; DF, degrees of freedom; LS, least squares; MFM32, 32-item Motor Function Measure; PGI-C, Patient/Parent Global Impression of Change.

## Discussion

This study provides evidence that the MFM32 total score is a valid, reliable and responsive assessment of motor function ability in individuals with Type 2 and non-ambulant Type 3 SMA. Specifically, supportive evidence of reliability was demonstrated by high ICCs (test-retest reliability) and a high Cronbach’s α (internal consistency). The magnitude and pattern of Spearman rank order correlation coefficients were consistent with expectations. While MFM32 scores at baseline exhibited strong correlations with both HFMS and FVC scores, correlation with HFMS was stronger than with FVC, providing evidence of convergent validity. Of note is the high correlation between the MFM32 and HFMS scores, consistent with the strong conceptual overlap between these scales. Furthermore, the MFM32 was able to discriminate between groups that were categorized by HFMS total or pre-specified item scores. Comparing baseline MFM scores between groups defined by HFMS scores (non-standers vs standers and sitters vs non-sitters) demonstrated a significant difference between groups, thus providing evidence of known-groups validity.

This study also provides evidence of the ability of the MFM32 to detect change. Despite the majority of patients being rated as “no change” on both the CGI-C and PGI-C, the MFM32 was still able to detect significant differences between those classified as stable or improving and those classified as worsening, as defined by these anchor scales.

Due to the retrospective nature of the study design, there are several limitations to consider when interpreting these findings. If prospectively designing a validation study, the selection and timing of assessments would differ to better fit the needs of psychometric analyses. For example, the HFMS was administered at screening rather than baseline, resulting in a variable gap, ranging from 1 to 4 weeks, between the rating of the HFMS and MFM32. Whilst this period of time is unlikely to reflect much difference in motor function ability, small changes cannot be discounted. This impacts the interpretation of both the convergent and known-groups validity results. For FVC, the height was estimated rather than measured, thus the FVC values should be considered estimates. The population includes a broad age range and, due to sample size limitations, no subgroup analyses were performed. Age effects are possible, given the inclusion of children, adolescents and adults. Additionally, these results do not provide evidence for children below 6 years or adults above 27 years. Finally, the PGI-C mixes patient and parent ratings. Differences between patients and parents have been reported on other scales in SMA [34]. Despite the limitations with the retrospective use of this data sample, the results still provide useful evidence of the MFM’s measurement properties.

This analysis complements the existing evidence, with findings consistent with those in a broader neuromuscular disease population, and prior analyses of convergent and known-groups validity in SMA [16, 17]. The MFM32 correlation with FVC in this study (ρ = 0.61) was a similar magnitude but lower than that identified by in a baseline analysis of the NatHis SMA study (ρ = 0.70). In this European, prospective, multicenter, longitudinal natural history study of Type 2 and 3 SMA, correlations with Myogrip and Myopinch (measures of upper arm strength) were similar to the correlation between MFM32 and HFMS identified in this analysis. These consistent findings provide additional confidence in use of the MFM32 to assess motor function in individuals with Types 2 and 3 SMA.

Future research gaps include establishing content validity and estimating meaningful within-patient change. Content validity is established by demonstrating that the items measure function that is meaningful to patients and their families. For example, in a study assessing the content validity of a related measure, the Expanded Hammersmith Functional Motor Scale, 55 individuals (30 parents and 25 patients) were able to relate all of the 33 items to activities of daily living [35]. Estimating meaningful within-patient change thresholds is critical for the interpretation of clinical outcome assessment data from interventional studies. A recent qualitative survey involving 822 respondents (including 436 patients and 370 parents), indicated that stabilization would be an important outcome of treatment for 96.5% of participants [36]. This finding is supported by qualitative research with patients and parents who reported that maintaining existing abilities is important, and that even small changes in function are meaningful [37]. In support of this qualitative evidence, in a suitable sample (e.g., in a dataset that includes both a suitable anchor measure, and a broad range of change in motor function), anchor- and distribution-based analyses should be conducted to estimate a meaningful within-patient change threshold to support interpretation of MFM32 data.

## Conclusions

Overall, these analyses provide robust supportive evidence of the validity, reliability and ability to detect change of the MFM32 in patients with Type 2 and non-ambulant Type 3 SMA. These results support the use of the MFM32 total score in longitudinal studies involving individuals with Type 2 and non-ambulant Type 3 SMA.
